# The Chemopreventive and Anticancer Potential of Glucosinolates and Their Hydrolysis Products from Cruciferous Vegetables

**DOI:** 10.3390/nu18050751

**Published:** 2026-02-26

**Authors:** Mateusz Labudda, Anna Rybarczyk-Płońska, Kamil Aleksander Sobieszek, Tomasz Niedziński, Wesley Borges Wurlitzer, Ewa Muszyńska, Beata Prabucka, Szymon Florczak, Monika Tomczykowa, Wojciech Makowski, Jakub Graska, Jakub Frankowski, Paulina Kęszycka, Danuta Gajewska, Abdelfattah A. Dababat, Iwona Morkunas, Joanna Trafiałek, Michał Tomczyk, Michał Czapla

**Affiliations:** 1 Department of Biochemistry and Microbiology, Institute of Biology, Warsaw University of Life Sciences—SGGW, Nowoursynowska 159, 02-776 Warsaw, Poland; anna_rybarczyk_plonska@sggw.edu.pl (A.R.-P.); beata_prabucka@sggw.edu.pl (B.P.); jakub_graska@sggw.edu.pl (J.G.); 2 Section of Plant Physiology and Biochemistry, Polish Botanical Society, Ujazdowskie 4, 00-478 Warsaw, Poland; ewa_muszynska@sggw.edu.pl (E.M.); wojciech.makowski@urk.edu.pl (W.M.); 3 Faculty of Medicine, Jagiellonian University Medical College, Jagiellonian University, Św. Anny 12, 31-008 Krakow, Poland; sobieszekkamil@gmail.com; 4 Division of Agricultural and Environmental Chemistry, Institute of Agriculture, Warsaw University of Life Sciences—SGGW, Nowoursynowska 159, 02-776 Warsaw, Poland; tomasz_niedzinski@sggw.edu.pl; 5 Laboratory of Acarology, Tecnovates, University of Vale do Taquari—Univates, Lajeado 95914-014, RS, Brazil; wesleeywurlitzer@gmail.com; 6 Postgraduate Program in Environment and Development, University of Vale do Taquari—Univates, Lajeado 95914-014, RS, Brazil; 7 Bioagro—Institute of Biotechnology Applied to Agriculture/INCT–Plant-Pest Interactions, Federal University of Viçosa—UFV, Viçosa 36570-900, MG, Brazil; 8 Department of Botany and Plant Physiology, Institute of Biology, Warsaw University of Life Sciences—SGGW, Nowoursynowska 159, 02-776 Warsaw, Poland; 9 Department of Organic Chemistry, Faculty of Medicine with the Division of Dentistry and Division of Medical Education in English, Medical University of Białystok, Mickiewicza 2a, 15-222 Białystok, Poland; monika.tomczyk@umb.edu.pl; 10 Department of Botany, Physiology and Plant Protection, Faculty of Biotechnology and Horticulture, University of Agriculture in Krakow, Mickiewicza 21, 31-120 Kraków, Poland; 11 School of Medical and Health Sciences, VIZJA University, Okopowa 59, 01-043 Warsaw, Poland; j.frankowski@vizja.pl; 12 Department of Dietetics, Institute of Human Nutrition Sciences, Warsaw University of Life Sciences—SGGW, Nowoursynowska 159, 02-787 Warsaw, Poland; paulina_keszycka@sggw.edu.pl (P.K.); danuta_gajewska@sggw.edu.pl (D.G.); 13 Soil Borne Pathogens Program, International Maize and Wheat Improvement Center (CIMMYT-Türkiye), Cem Erserver Caddesi No: 9-11 Yenimahalle, 06810 Ankara, Türkiye; a.dababat@cgiar.org; 14 Department of Plant Physiology, Faculty of Agriculture, Horticulture and Biotechnology, Poznań University of Life Sciences, Wołyńska 35, 60-637 Poznań, Poland; iwona.morkunas@up.poznan.pl; 15 Department of Food Gastronomy and Food Hygiene, Institute of Human Nutrition Sciences, Warsaw University of Life Sciences—SGGW, Nowoursynowska 159, 02-776 Warsaw, Poland; joanna_trafialek@sggw.edu.pl; 16 Department of Biology and Pharmacognosy, Faculty of Pharmacy with the Division of Laboratory Medicine, Medical University of Białystok, Mickiewicza 2a, 15-222 Białystok, Poland; michal.tomczyk@umb.edu.pl; 17 Division of Scientific Research and Innovation in Emergency Medical Service, Department of Emergency Medical Service, Faculty of Nursing and Midwifery, Wroclaw Medical University, Parkowa 34, 51-618 Wroclaw, Poland; michal.czapla@umw.edu.pl; 18 Group of Research in Care (GRUPAC), Faculty of Health Sciences, University of La Rioja, Calle Duquesa de la Victoria, 26006 Logroño, Spain; 19 Nursing Care and Education Research Group (GRIECE), Department of Nursing, University of Valencia, 46010 Valencia, Spain

**Keywords:** biofortification, cancer prevention, cruciferous vegetables, glucoraphanin, glucosinolates, indole-3-carbinol, isothiocyanates, Nrf2 signaling, sulforaphane

## Abstract

Background/Objectives: Glucosinolates (GSLs) from cruciferous vegetables (CVs), sulfur (S)- and nitrogen-containing compounds, are enzymatically hydrolyzed by myrosinase (EC 3.2.1.147) to yield bioactive derivatives such as isothiocyanates (ITCs) and indoles. These metabolites exhibit chemopreventive and anticancer properties. The article compiles evidence regarding the following: (i) the molecular mechanisms regulating the biosynthesis of key derivatives, including sulforaphane (SFN), phenethyl isothiocyanate (PEITC), and indole-3-carbinol (I3C); (ii) epidemiological and clinical findings; and (iii) strategies to link plant science with nutritional interventions for cancer prevention. Methods: An integrative literature review was conducted using Web of Science, Scopus, ScienceDirect, Google Scholar, and PubMed. English-language studies addressing mechanistic insights, nutritional factors, epidemiology, and clinical trials were included. Results: The biosynthesis and metabolism of GSL in plants are regulated by S and several transcription factors that promote or repress GSL production. Additionally, food processing has been shown to influence retention time and the formation of ITCs. In humans, ITCs activate nuclear factor erythroid 2-related factor 2 (Nrf2)-mediated detoxification, induce apoptosis, and modulate epigenetic pathways. Epidemiological data show inverse associations between CV intake and cancer risk, though variability exists. Clinical trials have confirmed the bioavailability and effects of glucoraphanin and SFN on cancer-related biomarkers. Conclusions: The described compounds are bioavailable in humans and modulate the clinically relevant pathways linked to carcinogenesis. Larger, standardized interventions are needed to determine effective intake levels, optimize bioavailability, and define their potential role in evidence-based nutritional strategies for cancer prevention.

## 1. Introduction

The Brassicaceae family, also referred to as Cruciferae, includes essential vegetables such as broccoli, Brussels sprouts, cabbage, cauliflower, collard greens, kale, mustard, and turnips. An increasing body of research underscores the significant influence of plant-based foods on human health [1,2,3,4,5]. Plant secondary metabolites, which have evolved as defense compounds [6,7,8], are garnering significant interest due to their potential to modulate various human diseases, including cancers [9,10]. Glucosinolates (GSLs), sulfur (S)- and nitrogen-containing compounds derived from amino acid precursors, exhibit considerable structural diversity, with more than 120 types classified into three categories: aliphatic, indolic, and aromatic [11,12,13,14,15]. These metabolites of cruciferous vegetables (CVs) are particularly noteworthy because when their tissues are disrupted, GSLs can be hydrolyzed by a myrosinase enzyme (MYR) (EC 3.2.1.147) to yield a variety of bioactive compounds, such as isothiocyanates (ITCs) and indoles, including sulforaphane (SFN), phenethyl isothiocyanate (PEITC), and indole-3-carbinol (I3C) [2,16,17,18,19,20,21,22,23,24,25].

The mentioned bioactive compounds may also be considered as antinutritional factors due to their capacity to form goitrogens. This concern primarily relates to disrupting the iodine uptake by the thyroid gland and inhibiting thyroid peroxidase activity. This disruption can potentially influence thyroid hormone synthesis and contribute to thyroid gland enlargement. However, a comprehensive systematic review published by Galanty et al. [26] provides compelling evidence that previous concerns regarding the significant antithyroid effects of CVs in humans have been exaggerated. According to this review, most available in vitro, in vivo, and human intervention studies suggest that moderate, regular consumption of CVs does not impair thyroid function, especially when iodine intake is adequate, and does not cause adverse changes in thyroid mass or hormone levels [26]. Furthermore, recent nutrition-focused analyses indicate that their physiological impact is significant only in cases of excessive or atypical consumption or concurrent iodine deficiency. Under typical dietary patterns, the overall health benefits of consuming CVs clearly outweigh any potential antinutritional effects. A balanced diet and sufficient iodine intake effectively minimize the physiological risks posed by the goitrogenic compounds found in these vegetables, reinforcing the conclusion that CVs remain a safe and valuable component of the diet even for thyroid health when consumed in reasonable amounts [26,27].

Broccoli sprouts have garnered considerable attention as a functional food, characterized by their high concentrations of glucoraphanin (GRA), a stable GSL precursor of SFN, which are significantly higher than those in mature broccoli. In addition to GRA, these sprouts contain a range of phytochemicals, including phenolic acids, flavonoids, and carotenoids, all of which possess strong antioxidant properties. Recent research highlights the bioactivities of these phytochemicals, indicating that broccoli sprout extracts are rich in GRA and SFN, as well as sinapic and caffeic acids, lutein, zeaxanthin, quercetin, and kaempferol. Furthermore, these extracts exhibit notable in vitro activities, including free radical scavenging, metal chelation, and lipoxygenase inhibition, thereby establishing their significance as a bioactive-rich dietary source of ITCs and complementary antioxidants [28]. Recent long-term clinical evidence has emerged, particularly from a 42-month randomized, double-blind, placebo-controlled pilot trial focusing on older adults at an elevated risk of dementia, including individuals diagnosed with a mild cognitive impairment. This trial demonstrated that daily supplementation with GRA derived from broccoli sprout capsules (which include active MYR) resulted in significantly improved Memory Performance Index scores over time compared with the placebo group (group × time interaction; *p* = 0.012). Urinary measurements of the GRA metabolite, SFN-N-acetyl-L-cysteine (SFN-NAC), confirmed the exposure without raising significant safety concerns, suggesting potential long-lasting neurocognitive benefits from ITCs sourced from these sprouts, particularly when incorporated into regular dietary routines [29]. Moreover, advancements in food technology are enhancing the methods to maximize the bioactivity of these sprouts from production to consumption. A study by Yu et al. [30] reported that pre-harvest sucrose stress substantially increases GRA production in early sprouts, achieving an approximate 316% increase compared to the control group by day 4. Additionally, vacuum freeze drying these sprouts yields a shelf-stable powder that retains a significant amount of SFN (approximately 6.0 mg/g dry weight), along with a robust total ITC content and advantageous techno-functional properties, including excellent water- and oil-holding capacities. This presents a feasible solution for standardizing high-content sprout ingredients for nutritional and clinical applications [30]. In summary, these converging factors, analytical profiling, human efficacy, and process optimization, position broccoli sprouts as a rich, scalable, and clinically relevant source of GSL-derived ITCs, thereby paving the way for promising dietary strategies to prevent chronic diseases and promote healthy aging.

Epidemiological evidence consistently indicates a protective association between the consumption of CVs and a reduced risk of cancer; however, the effects appear to vary by cancer type, population, and the level of dietary exposure. A systematic review and dose–response meta-analysis by Zheng et al. [31] synthesized findings from 226 case–control and cohort studies published between 1978 and June 2023 to clarify the quantitative relationship between CV intake and various types of cancer. The pooled analysis revealed that the regular consumption of CVs is linked to a decreased overall cancer risk (odds ratio = 0.77; risk ratio = 0.96). The optimal protective intake levels varied across cancer types, ranging from approximately three servings per week for prostate cancer to over seven for gynecological cancers. Regional differences were also observed: intake was strongly correlated with a reduced risk of lung, head and neck, and esophageal cancers in Asian populations, while associations with colorectal, renal, gynecological, and prostate cancers were more pronounced in American cohorts. These findings highlight the multifactorial nature of the chemopreventive effects of CVs and underscore the significance of intake dose, frequency, and population context in determining their cancer-protective potential [31]. Recent research has uncovered both opportunities and challenges associated with the use of GSLs as chemopreventive agents. Studies consistently show that metabolites derived from GSLs activate protective cellular pathways and inhibit cancer cell proliferation. Although translational and clinical research are less extensive, they provide promising evidence supporting their effectiveness in reducing the risk of several cancers, particularly colon, breast, and prostate cancers [32,33,34,35,36]. Moreover, advances in plant sciences have enabled the manipulation of GSL biosynthesis and accumulation, with the results increasingly indicating that biotechnology factors and postharvest processing significantly influence GSL profiles in CVs. Thus, the dynamic interface between food technology, nutrition, and health-oriented applications underscores the importance of interdisciplinary perspectives in addressing challenges such as cancer prevention [37,38,39,40].

This article specifically addresses the following areas: (1) the bioactive compounds that have consistently demonstrated chemoprotective and anticancer properties in preclinical studies; (2) the breadth and limitations of clinical and epidemiological evidence supporting the role of analyzed compounds in cancer prevention and treatment; and (3) the potential for advancements in plant science and biotechnology to enhance the translational relevance of these bioactive compounds. An integrative review was conducted to gather the pertinent literature, employing a structured search methodology complemented by critical analysis. The literature search was conducted between May 2025 and February 2026 across various scientific databases, including Web of Science, Scopus, ScienceDirect, Google Scholar, and PubMed. The primary keywords utilized in this search encompassed “glucosinolates”, “Brassicaceae”, “cruciferous vegetables”, “bioactive compounds”, “nutritional properties”, “anticancer properties”, “chemopreventive properties”, “isothiocyanates”, “sulforaphane”, “phenethyl isothiocyanate”, and “glucoraphanin”, in addition to other related terms. The inclusion criteria for this review were intentionally restricted to English-language research articles, thereby ensuring a high standard of scientific quality and relevance. Consequently, materials such as popular science articles, theses, and editorials were excluded from consideration. Following an initial screening, a thorough content analysis was conducted to prioritize articles that provide empirical data, theoretical insights, or significant advancements in plant science, human nutrition, translational medicine, and the application of CVs in cancer treatment. Although the methodology employed does not strictly conform to that of a systematic review, it was purposefully designed to provide a thorough and representative overview of the existing literature. The emphasis was placed on investigating the potential role of CVs in targeted nutrition and dietary therapy for specific cancers and demographic groups.

In light of the rising global incidence of cancer, research focusing on CVs underscores the significant potential these plants may hold. Consequently, the selection of this topic, area of inquiry, and time frame was made with careful consideration to establish a literature review that can serve as a valuable foundational reference for future research initiatives. This review presents a synthesis of current evidence on the factors influencing GSL biosynthesis in plants, bioavailability, and chemopreventive and anticancer activities, integrating insights from biochemical and clinical research. Although its integrative approach enhances the scope of analysis, certain limitations must be acknowledged. As a narrative review, it is inherently vulnerable to selection bias. Additionally, the variability of glucosinolate metabolism among different genotypes and populations, the focus on major Brassicaceae species, and the scarcity of large-scale, randomized controlled trials can limit the generalizability and accuracy of some findings. Moreover, the dynamic interactions between plants and their environments along with the evolving body of clinical evidence may require periodic updates to ensure ongoing relevance and precision. In light of these considerations, this review has been organized to reflect the current state of knowledge in the field.

## 2. Glucosinolate Biosynthesis and Determinants of Accumulation in Plants

### 2.1. Biosynthesis

**Figure 1:**
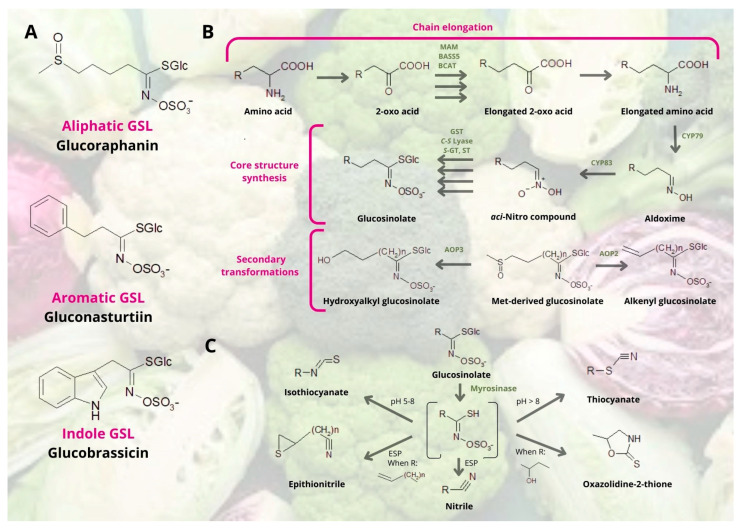
Classes of glucosinolates (GSL), their biosynthetic pathways, and enzymatic hydrolysis products [43,44]. (**A**) Representative structures of the three major GSL classes: aliphatic, aromatic, and indole. (**B**) Simplified scheme of GSL biosynthesis, including amino acid chain elongation, core structure formation, and secondary modifications, with selected enzymes involved in methionine-derived GSLs. (**C**) Glucosinolate hydrolysis upon tissue disruption and the formation of major breakdown products catalyzed by myrosinase, depending on substrate structure and reaction conditions. MAM: methylthioalkylmalate synthase; BASS5: bile acid: sodium symporter family protein 5; BCAT: branched-chain aminotransferase; GST: glutathione *S*-transferase; *S*-GT: *S*-glucosyltransferase; ST: sulfotransferase; AOP: α-ketoglutarate dioxygenase; and ESP: epithiospecifier protein.

The biosynthesis of GSLs in plants is influenced by the selection of amino acid precursors specific to various subclasses of GSLs (Figure 1). In general, methionine, valine, leucine, or isoleucine precursors give rise to most aliphatic GSLs, tryptophan gives rise to indolic GSLs, and phenylalanine or tyrosine to aromatic GSLs. However, Blazević et al. [41] suggest that a GSL’s appropriate classification should be based solely on its amino acid precursor since indole GSLs are also aromatic. At the same time, Wang et al. [23] and Kattel and Antonious [42] propose a better understanding of these commonly accepted three subclasses for tissue-specific GSL accumulation. Simplified GSL synthesis, divided into three stages, is presented in Figure 1 and includes GSL chain elongation, core structure synthesis, and secondary modifications [43,44].

The chain elongation step, relevant for precursors of some aliphatic and aromatic GLS, begins with the deamination of the parent amino acid to form the corresponding 2-oxo acid (Figure 1). Next, the 2-oxo acid undergoes a three-step transformation to yield an elongated 2-oxo acid, which can enter either the second round of chain elongation or be subjected to transamination to form an elongated amino acid. In the amino acid chain elongation step of methionine-derived GSLs, methylthioalkylmalate synthase (MAM), a bile acid: sodium symporter family protein 5 (BASS5), and a branched-chain aminotransferase (BCAT) are involved. The core structure synthesis begins with two oxidation reactions catalyzed by cytochromes P450 belonging to the CYP79 and CYP83 families. The unstable product, an aci-nitro compound, further undergoes a four-step transformation, catalyzed by glutathione S-transferase (GST), C-S lyase, S-glucosyltransferase (S-GT), and sulfotransferase (ST), respectively. The possible secondary transformations of the R group of GSL include oxidation, elimination, alkylation, or esterification. Here (Figure 1), two common oxidative secondary transformations of methionine-derived GLS catalyzed by α-ketoglutarate dioxygenases AOP2 and AOP3 are presented. The GSL hydrolysis and its products are also presented (Figure 1). Upon plant damage (e.g., when chopping or chewing in the mouth), the GLSs are hydrolyzed by β-thioglucoside glucohydrolase, which is localized in myrosin cells and which is called MYR. The unstable aglucone intermediate, shown in the bracket, further rearranges to form either isothiocyanates, thiocyanates, epithionitriles, nitriles, or oxazolidine-2-thiones, depending on the R group structure, pH, or the presence of specific proteins or cofactors, including epithiospecifier protein (ESP) (Figure 1) [43,44].

The influence of myeloblastosis transcription factors (MYBs) on the expression of genes involved in both the core synthesis and side chain modifications determines the production of different types of GSLs. Sulfur metabolism pathways supply the S backbone of the GSL structure and thus are critical for regulating GSL levels, which, along with MYB-regulated pathways, are determined by these pathways. According to Kattel and Antonious [42], integrated research examining how S assimilation influences the transcriptional control of GSLs is vital to developing crop production strategies that enhance chemopreventive roles and agricultural productivity. Moreover, advances in heterologous expression systems have enabled biochemical analyses of the enzyme systems involved in GSL biosynthesis and hydrolysis. The characterization of substrate specificities for each of the Arabidopsis MYR isoforms, AtTGG1, AtTGG4, and AtTGG5, when introduced into the yeast system, enables modeling studies for predicting enzyme activity [23].

### 2.2. Factors Regulating Glucosinolate Levels from Plant Growth to Food Processing

Knowledge of tissue-specific GSL content across developmental stages can help in the design of harvest strategies for CVs to support chemoprotection. The expression of GSLs in plants’ edible portions can vary widely across cultivars, tissues, and harvest times. For example, methylsulfinylalkyl GSLs are found in high concentration in young broccoli sprouts, while indolic GSLs predominate in mature broccoli tissues [45]. Significant changes in GSL accumulation also occur as a consequence of breeding approaches. Bussler et al. [35] suggest that broccoli lines selected for high GRA content can increase antiproliferative activity by 44% compared to commercial cultivars. Based on the in vitro IC_50_ values of the glucosinolate mixture components, the mixture model in breeding can be used to develop cultivars with maximal anticancer potency rather than just total GSL levels.

The study conducted by Kushad et al. [46] investigated 65 accessions of CVs grown under uniform conditions, revealing significant diversity in both the types and concentrations of GSLs within and across the groups. Notably, broccoli displayed considerable variability, with its GSL profile predominantly consisting of GRA, gluconapin (GNA), and glucobrassicin (GBS), and GRA levels ranging from 0.8 to 21.7 μmol g^−1^ dry weight. In contrast, Brussels sprouts, cabbage, cauliflower, and kale were primarily characterized by sinigrin and GBS, with Brussels sprouts also exhibiting substantial levels of GNA. The marked differences in GSL content underscore the strong genotype dependence, suggesting valuable opportunities for breeding or genetic enhancement to improve the health-promoting attributes of these vegetables.

Environmental factors, including edaphic conditions such as pH levels, water availability, nutrient concentrations, and various abiotic and biotic stresses, significantly impact the overall crop yield and the specific concentrations of GSLs within crops. For example, Chinese cabbage grown in soils with a high pH value can contain higher levels of gluconasturtiin and GNA than plants grown in soils with a low pH. Turnips with increased GSL concentration are obtained under limited irrigation practices. Phosphorus and light manipulation studies suggest that a limited phosphorus supply, combined with optimal light exposure, can increase the total GSLs by over 160% [42]. Furthermore, salt treatments applied to Chinese cabbage plants have been found to reduce aliphatic GSL concentrations under high NaCl and KCl, and to increase indolic and aromatic GSL levels upon treatment with Na_2_SO_4_ [47]. Different GSLs also accumulate differentially between seasons. Spring-sown red cabbage plants contain lower GSL levels as compared to fall-sown cabbage plants [48]. There is also positional regulation of GSLs in the plant. For example, the inner leaves of cabbage had a higher GSL concentration compared to the outer leaves [48]. In red and green cabbage, different types of GSLs are found, resulting in widely variable concentrations [45].

The study by Brown et al. [49] examined the GSL profiles of ten broccoli accessions grown across various environments to better understand how genotype, environment, and their interactions contribute to variation in these compounds. The researchers identified significant genetic variability in aliphatic GSLs, with genotype accounting for over half of the variation in key compounds such as GRA and progoitrin, whereas indolyl GSLs showed considerably less genetic influence. They also noted dramatic differences up to ten-fold in levels of major aliphatic GSLs among the accessions, with only two lines producing notable amounts of GSLs beyond GRA. Seasonal planting further highlighted variations in GSL stability, underscoring the interactions between genetics and environmental conditions. Overall, these findings indicate that the existing broccoli germplasm possesses the genetic potential to modify GSL profiles, thereby supporting the feasibility of breeding broccoli cultivars with enhanced cancer-protective properties.

Postharvest handling and storage are two crucial steps in maintaining the nutritional value of CVs. It has been reported that the cold storage of broccoli for 7 days either maintains the same GSL content or even increases it, whereas storage at higher temperatures or for a longer period results in a dramatic decrease in GSL content. Moderate light exposure after 3 days of cold storage at 10 °C can increase GSL levels to levels even higher than those at harvest in broccoli. The flower buds of broccoli accumulated GSL content up to 130% higher than the at harvest time under the same conditions [50].

Different cooking practices play an important role in the retention and bioavailability of GSLs and their derivatives in CVs. Boiling broccoli for 10 min decreases GSL by up to 57% and also reduces isothiocyanate bioavailability to approximately one third of that of raw, steamed, or stir-fried broccoli [47]. Controlled heating up to 50 °C has also been shown to be optimal for retaining SFN, while an 80 °C treatment preserves the GSL content [21]. Both biofortification strategies that increase the overall concentration of health-promoting GSLs and elicitation strategies that increase metabolite levels in sprouts and microgreens through stress imposition [34] can be implemented for optimal postharvest handling. Therefore, postharvest biotechnology can be coupled with breeding and agronomic practices. According to recent findings, evidence-based guidelines for the postharvest handling and cooking of these vegetables are urgently needed to enable the human population to benefit from the anticancer effects of GSLs.

## 3. Anticancer Properties, Mechanisms, and Metabolic Pathways of Glucosinolate-Derived Metabolites

**Figure 2:**
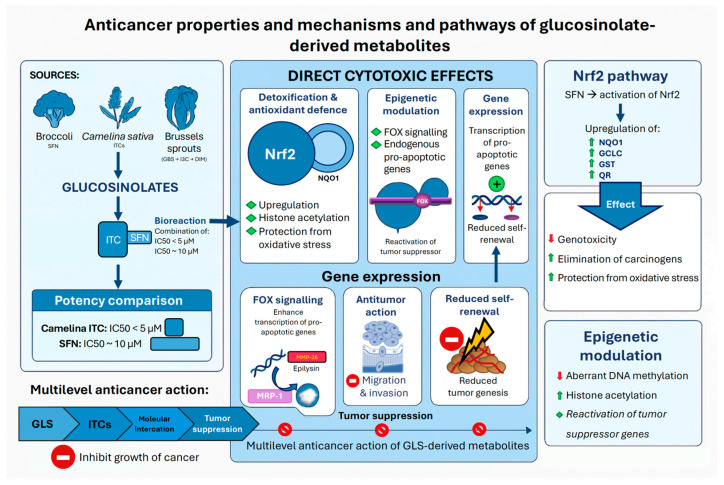
Anticancer properties, mechanisms, and metabolic pathways of glucosinolate-derived metabolites. The diagram illustrates the examples of plant sources of glucosinolates (e.g., broccoli, *Camelina sativa*, and *Brussels sprouts*) and their bioconversion into bioactive metabolites, such as isothiocyanates (ITCs) and sulforaphane (SFN). These compounds exert multilevel anticancer effects, including direct cytotoxic activity, the activation of the nuclear factor erythroid 2-related factor 2 (Nrf2) pathway, epigenetic modulation, and the regulation of gene expression. Together, these mechanisms strengthen antioxidant defenses, promote the upregulation of pro-apoptotic genes, restore the activity of tumor suppressor genes, inhibit cancer cell migration and invasion, and reduce the self-renewal capacity of malignant cells. Overall, the figure illustrates the integrated, multi-target anticancer actions of glucosinolate (GLS)-derived metabolites. GBS: glucobrassicin; I3C: indole-3-carbinol; DIM: 3,3′-diindolylmethane; NQO1: NAD(P)H quinone dehydrogenase 1; FOX: forkhead box; GCLC: glutamate-cysteine ligase catalytic subunit; GST: glutathione *S*-transferase; QR: quinone reductase; MRP-1: multidrug-resistance-associated protein-1; and MMP-28: matrix metalloproteinase 28.

Glucosinolate-derived metabolites, including SFN and PEITC, along with other bioactive ITCs, exert a range of anticancer effects through targeted, cancer-selective mechanisms (Figure 2).

For example, SFN has been shown to reduce cell viability, induce G0/G1 phase arrest, and trigger apoptosis in colorectal and lung cancer models, underscoring its ability to inhibit the proliferation of cancer cells (Figure 2). Additionally, it exhibits dose- and time-dependent apoptosis rates in human A549 cancer cells derived from lung tissue, specifically alveolar epithelium, as well as in the human colorectal cancer cell lines HT-29, indicating its selective cytotoxicity against malignancies [51,52]. In comparison, camelina-derived ITCs exhibit higher potency in lung cancer cell models than SFN, with an IC_50_ of <5 µM for camelina ITC and ~10 µM for SFN. This roughly two-fold cytotoxic superiority of camelina ITC over SFN highlights the importance of ITC’s specific chemical features [52]. The anticancer mechanisms of GLS derivatives from Chinese cabbage, mainly aliphatic and indole ITCs, also exhibit pro-apoptotic and cytotoxic effects in human hepatocellular carcinoma (HepG2) liver cancer cells [53]. The effectiveness of these glucosinolate-derived compounds in Chinese cabbage is significant, emphasizing the biological and functional relevance of GLS diversity across cruciferous crops for cancer prevention. The rapid antiproliferative response to these metabolites, which irreversibly inhibit cancer cell growth within 3 h of treatment, underscores their clinical potential (Figure 2) [54].

Sulforaphane can activate and enhance various chemoprotective pathways that defend against carcinogens and oxidants in cell cultures. It effectively activates the nuclear factor erythroid 2-related factor 2 (Nrf2) signaling pathway, which upregulates phase II detoxification enzymes, such as quinone reductase (QR) and glutathione *S*-transferase (GST). The upregulation of detoxifying enzymes facilitates the elimination of carcinogenic compounds, decreases genotoxicity, and prevents or mitigates oxidative damage, indicating that these phase II enzymes play a crucial role in the protective effects of SFN [55]. Sulforaphane also increases the activity of QR and GST in various cell culture models, indicating its effectiveness in detoxification. Furthermore, the reduction in cancer stem cell markers, such as Nanog, Oct4, Sox2, and ΔNp63α, confirms the important role of SFN in inhibiting colorectal cancer stem cell self-renewal, thereby reducing tumor growth (Figure 2) [56].

Aside from activating detoxifying enzymes, SFN also alters the expression of other genes involved in apoptosis, cell survival, and transcription factors, such as the mammalian forkhead class O (FOXO) transcription factors [57,58]. In addition, SFN and other ITCs can promote the degradation of pro-malignant genes (including those involved in migration, invasion, and metastasis, such as matrix metalloproteinase 28 (MMP-28), also known as epilysin) or activate tumor suppressor genes [59]. By inducing G0/G1 cell cycle arrest in cancer cells, these metabolites can delay tumorigenesis [38] and have been shown to exert epigenetic effects, such as altered DNA methylation patterns and histone acetylation, which may restore the function of silenced tumor suppressor genes [54]. Glucosinolate-derived metabolites have demonstrated sustained efficacy against multidrug-resistant cancer cells characterized by the overexpression of efflux proteins, specifically multidrug-resistance-associated protein-1 (MRP-1) and P-glycoprotein-1. These metabolites exhibit notable anticancer activity in both sensitive and multidrug-resistant cell lines. In vitro studies indicate that ITCs, along with their glutathione conjugates, possess significant anticancer properties (Figure 2) [54].

Chronic exposure to carcinogens is a significant risk factor for head and neck squamous cell carcinoma (HNSCC). Sulforaphane from broccoli sprout extracts (BSE) has been shown to enhance the detoxification of airborne pollutants in humans. Bauman et al. [60] explored the chemopreventive potential of SFN using both in vitro and in vivo models of oral carcinogenesis. The treatment of normal mucosal epithelial cells (Het-1A) and HNSCC cell lines with SFN resulted in the activation of NRF2 and the upregulation of its target genes, *NAD(P)H quinone dehydrogenase 1* (*NQO1*) and *glutamate-cysteine ligase catalytic subunit* (*GCLC*), in a dose- and time-dependent manner. Additionally, SFN inhibited oncogenic signaling by promoting the NRF2-independent dephosphorylation of pSTAT3, a key oncogenic factor in HNSCC. In a murine model of oral cancer induced by 4-nitroquinoline-1-oxide (4NQO), SFN administration significantly decreased both tumor incidence and size. A pilot clinical study involving ten healthy volunteers compared the bioavailability and mucosal bioactivity of various BSE formulations. The SFN-rich BSE exhibited the highest and most consistent bioavailability, while mucosal NQO1 transcript levels were upregulated in several participants across all treatment regimens. Together, these findings provide preclinical and early clinical evidence supporting SFN as a promising chemopreventive agent against carcinogen-induced oral cancer and tobacco-related HNSCC (Figure 2) [60].

Hypoxia-induced carbonic anhydrase IX (CA IX) is a clinically validated target for anticancer therapy, yet there is a scarcity of naturally occurring, isoform-selective inhibitors. In a study by Kamel et al. [61], GRA, 1,4-dimethoxyglucobrassicin, and 4-methoxyglucobrassicin (4-MeO-GB) were extracted from broccoli and assessed for their CA IX inhibitory potential using a comprehensive experimental and computational approach. Enzymatic assays demonstrated mixed-type inhibition with nanomolar potency (IC_50_ = 65–221 nM) and significant selectivity over the housekeeping isoforms CA I and CA II (greater than 40-fold). Molecular docking, in conjunction with 500-nanosecond molecular dynamics simulations, free energy landscape mapping, and the molecular mechanics Poisson–Boltzmann surface area (MM-PBSA) analysis, consistently identified 4-MeO-GB as the most potent ligand. This compound was characterized by deep hydrophobic insertion, stable hydrogen bonding, and favorable van der Waals and electrostatic interactions (Δ_GMM/PBSA_ = −25 kJ mol^−1^). In silico, the absorption, distribution, metabolism, excretion, and toxicity profiling indicated low risks associated with the human ether-a-go-go-related gene and cytochrome P450, but suggested limited oral absorption and moderate hepatotoxicity, underscoring the necessity for targeted delivery strategies or scaffold refinement. Overall, 4-MeO-GB emerges as a promising natural scaffold for the selective inhibition of CA IX, providing valuable insights for structure-guided drug design against hypoxia-associated cancers (Figure 2) [61].

Glucobrassicin, an indole glucosinolate abundant in CVs, is metabolized to I3C, a compound with established anticancer properties in preclinical studies. In a clinical trial conducted by Fujioka et al. [62], the quantitative relationship between GBS intake and the urinary excretion of its metabolite, 3,3′-diindolylmethane (DIM), was investigated. Forty-five participants consumed Brussels sprouts and/or cabbage and received seven graded doses of GBS (25–500 μmol) once daily for two consecutive days, with complete 24 h urine collections after each dose. Urinary DIM levels, quantified by LC/ESI-MS/MS-SRM, increased proportionally with GBS intake and plateaued between 200 and 300 μmol. The correlation between the GBS dose and urinary DIM was strong (R^2^ = 0.68), and most DIM was excreted within 12 h of consumption. These findings demonstrate that urinary DIM serves as a robust biomarker of GBS exposure and I3C uptake, and that increasing GBS intake beyond 200 μmol does not enhance DIM excretion, indicating a plateau in its potential chemopreventive response (Figure 2) [62].

Consistent with these experimental studies, emerging evidence indicates that CV consumption plays a protective role in cancer etiology. It has been suggested that a high consumption of CVs may reduce the risk of cancer [63]. In a prospective cohort study, individuals who consumed high amounts of CVs demonstrated significant reductions in relative risk for colon cancer and breast cancer, with values of 0.67 and 0.63, respectively. Furthermore, those who regularly consumed CVs were found to have a reduced risk of colon cancer, rectal cancer, breast cancer, and lung cancer, with a decrease of approximately 40–50% compared to the control group [64]. Systematic reviews indicate that two-thirds of all case–control studies demonstrate a negative correlation between the consumption of CVs and the risk of cancer. This finding is further corroborated by observations that isothiocyanate-elevated urinary levels are associated with a reduced risk of lung cancer [65]. In broccoli sprouts, which contain SFN, some randomized controlled trials suggest that they modulate biomarkers relevant to carcinogenesis, such as decreased mRNA expression of α-methylacyl-CoA racemase in prostate cancer patients and increased hepatic GST activity [66]. However, evidence regarding the effects of CVs consumption on overall survival after a cancer diagnosis remains limited. Taken together, GLS-derived metabolites demonstrate broad potential as anticancer agents with multiple mechanisms (Figure 2).

## 4. Translational Evidence from Clinical Studies

**Figure 3:**
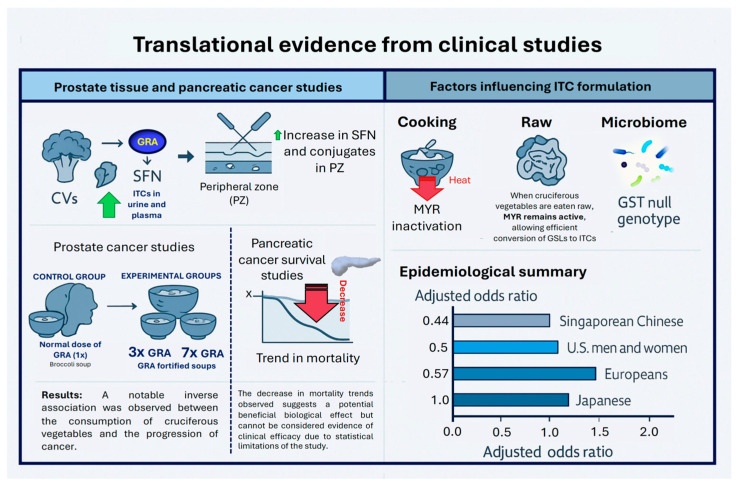
Translational evidence from clinical studies on glucosinolate-derived metabolites and isothiocyanates (ITCs). The figure summarizes key clinical and translational findings demonstrating the biological effects of ITCs, particularly sulforaphane (SFN), in human studies. In investigations of prostate and pancreatic cancer, the consumption of cruciferous vegetables (CVs) and glucoraphanin (GRA)-fortified foods increased SFN and its conjugates in tissues and bodily fluids, and was associated with reduced cancer progression and downward trends in mortality. Factors affecting ITC formation, such as cooking (which inactivates myrosinase, MYR) and the consumption of raw vegetables (which preserves MYR activity), as well as interindividual differences in microbiome composition or glutathione *S*-transferase (GST) null genotype, critically influence bioavailability and metabolic outcomes. Epidemiological data show an inverse relationship between cruciferous vegetable intake and cancer risk across diverse populations, with adjusted odds ratios indicating varying degrees of protective effects.

Controlled clinical trials have shown that GSLs derived from CVs, along with their metabolites, are bioavailable and can influence cancer-related biomarkers. This highlights the potential for enhancing the nutritional value of CVs through biofortification. Studies indicate that dietary supplement interventions containing SFN or its precursor, GRA, increase levels of ITC metabolites in both urine and plasma. This suggests that SFN is effectively absorbed and processed by the human body. Research shows that individuals who consume SFN excrete significantly higher levels of its metabolites in their urine than those who receive a placebo. However, it is essential to improve and carefully standardize preparation, delivery, and extraction methods in future studies [66,67,68]. In a study by Livingstone et al. [69], the aim was to investigate whether GRA supplementation, derived from broccoli seeds, results in the presence of bioactive compounds in prostate tissue. They conducted a four-week randomized, double-blind, 2 × 2 factorial dietary intervention involving 42 men scheduled for transperineal prostate biopsy, with 39 participants successfully completing the study. The active supplement used was BroccoMax^®^ (Jarrow Formulas, Los Angeles, CA, US), which contains GRA. The analysis of the prostate biopsy samples revealed significantly increased levels of SFN and its thiol conjugates (*p* < 0.0001) in both the transition and peripheral zones of the prostate among the men who received GRA supplements (Figure 3).

In a study conducted by Traka et al. [70], the impact of consuming GRA-rich broccoli soup on gene expression in the prostate tissue of men with localized prostate cancer over a one-year period was explored (Figure 3). The study involved 49 participants who underwent active surveillance and participated in a 12-month, randomized, double-blind dietary intervention with three groups. Each week, they consumed 300 mL of broccoli soup made from either standard broccoli (control) or two experimental genotypes with GRA levels 3- and 7-fold higher, respectively. Prostate tissue samples were taken before and after the intervention for RNA sequencing and gene set enrichment analyses. In the control group, several hundred changes in gene expression were observed in the non-neoplastic tissue throughout the year, which were linked to the upregulation of oncogenic pathways, including those related to inflammation and epithelial–mesenchymal transition. Conversely, these changes and pathway activations were reduced in a dose-dependent manner among participants who consumed the GRA-enriched broccoli soups. While the study lacked sufficient power to draw strong conclusions with regard to clinical outcomes, a notable inverse association was observed between the consumption of CVs and the progression of cancer. These findings suggest that the long-term consumption of GRA-rich broccoli soup may positively influence prostate gene expression, potentially lowering the risk of cancer progression (Figure 3) [70].

Recent studies suggest that daily administration of SFN and GRA may confer survival benefits in patients with pancreatic cancer (Figure 2) [66,71]. Although these trials suggest a reduction in mortality rates associated with these compounds, the findings did not reach statistical significance [66]. This limitation could be attributed to the relatively small sample sizes in the clinical trials. It is crucial to recognize that pancreatic cancer is one of the most aggressive forms of cancer, and it carries a poor prognosis, emphasizing the pressing need for additional clinical research. Such investigations are vital to assess whether dietary interventions utilizing derivatives of cruciferous GSLs can yield meaningful clinical benefits.

The effectiveness of interventions using CVs for cancer prevention largely depends on the metabolism of intact GSLs into active ITCs, as GSLs themselves lack bioactivity. When CVs containing MYR are consumed, this conversion occurs within plant cells during maceration, as cell structures break down. However, it is crucial to understand that the MYR naturally found in these plants becomes deactivated by heat. This means that when CVs are cooked, MYR is no longer available to facilitate the metabolism of GSLs (Figure 2). For instance, many Europeans typically cook CVs before eating, thereby inactivating endogenous MYR during cooking. In contrast, when these vegetables are served raw, as is customary in many Asian dishes, the MYR stays active, enabling the plant to exert its beneficial effects through ITCs. Consequently, the protective benefits provided by ITCs are only effective if the CVs are properly chopped or broken down [68,72]. Several studies suggest that hydrolyzed ITCs are absorbed more efficiently into the bloodstream than whole, uncooked GSLs, resulting in higher concentrations excreted from the body. The metabolic conversion to the active ITC can vary based on individual differences and the specific types of ITCs involved [68]. Thus, while dietary strategies incorporating cruciferous crops and their metabolites show promise, they also entail several limitations and challenges (Figure 3).

Diet alone does not determine the availability of ITCs; several factors contribute to the conversion of intact GSLs to ITCs. These factors include the composition of the microbiome and individual genetic variations [68]. Gene–environmental interactions can also alter how GSLs are metabolized and how ITCs are processed in a person’s body [73]. For example, individuals with a GST null genotype may experience greater reductions in cancer risk from cruciferous foods and GSLs than those with a functional GST gene, particularly for cancer risks associated with genes involved in detoxifying xenobiotics [74]. Thus, differences among populations, including genetics, preferred crop types, and even the timing of interventions relative to cancer onset, can complicate the results of these studies (Figure 3).

Research using in vitro models, such as cell cultures, has demonstrated that nanostructures, including polymeric micelles and solid lipid nanoparticles, can effectively deliver SFN and may contribute to cancer prevention [71]. Due to SFN’s low stability, lipophilicity, and poor water solubility, its bioavailability is often compromised. Therefore, encapsulating SFN within nanostructures is a promising strategy to enhance its delivery and therapeutic potential in cancer treatment [71]. Additional research is needed to evaluate the effectiveness of GSLs as cancer therapeutics and to explore ways to boost their bioactivity by improving oral bioavailability, particularly through innovative nanotechnology approaches (Figure 3).

Epidemiological studies, including case–control and cohort analyses, have provided compelling evidence suggesting a connection between a high consumption of CVs and a lower risk of colorectal cancer. Numerous studies indicate that individuals who consume the highest levels of these vegetables experience significantly reduced odds of developing colon cancer. For instance, the adjusted odds ratios are 0.44 for Singaporean Chinese populations, 0.50 for both U.S. men and women, and 0.57 for Europeans. However, a separate cohort study focusing on the Japanese population did not observe a similar link between CVs and colon cancer risk. This variation underscores the need for additional epidemiological research to better understand how these vegetables contribute to cancer risk in that specific demographic. The discrepancies noted across studies worldwide can largely be attributed to differences in regional dietary practices, genetic backgrounds, and the specific crop varieties consumed in each region. While research suggests a positive correlation between the intake of CVs and colon cancer prevention across several cohorts in North America, China, and Italy, many studies conducted in Europe indicate that these vegetables may be less effective or show no protective benefit at all (Figure 3) [75,76,77,78].

As can be concluded from the entire above argument, this review extends beyond previous descriptive studies by highlighting the complex interactions among genotype, environmental factors, and postharvest biology in determining glucosinolate levels and their effectiveness. By merging plant biotechnology with clinical research, a pathway is established that connects optimal crop practices with improved human health, underscoring the role of horticultural strategies in disease prevention. However, several limitations must be considered. The accumulation of GSLs is heavily influenced by environmental conditions, which impact reproducibility in field settings. Additionally, variations in individual genetics, metabolism, and microbiome composition can impact the bioavailability and efficacy of GSLs. Currently, clinical trials in this area are limited in size and duration, highlighting the necessity for more standardized, long-term, randomized controlled studies to validate health claims and establish evidence-based dietary recommendations.

## 5. Conclusions

Cruciferous vegetables are regarded as a highly promising source of dietary chemopreventive phytochemicals, primarily attributable to their GSL content and the biologically active hydrolysis products, such as ITCs and indoles, that they produce. As elaborated throughout this review, these compounds are produced through intricate plant metabolic pathways that are influenced by genetic, environmental, agronomic, and postharvest factors. These determinants ultimately shape the profile and concentration of health-relevant metabolites available for human consumption. Significant advancements in plant biotechnology, targeted breeding, optimized cultivation techniques, and enhanced storage and processing practices present substantial opportunities to increase glucosinolate accumulation, thereby potentially amplifying the anticancer efficacy of CVs.

Moreover, extensive experimental evidence from cellular and animal models consistently demonstrates the capacity of ITCs and indole derivatives to modulate critical molecular pathways involved in detoxification, oxidative stress response, apoptosis, cell cycle regulation, inflammation, and epigenetic modification. These multifaceted effects contribute to their efficacy in suppressing tumor initiation, progression, and metastasis. Epidemiological studies, although variable across populations, generally suggest an inverse correlation between the consumption of cruciferous vegetables and the incidence of various cancers, including colorectal, lung, breast, and prostate cancers. Additionally, translational studies and early-phase clinical trials further validate the bioavailability of GSL-derived metabolites in humans and their capacity to influence cancer-related biomarkers in target tissues. This reaffirms the significance of dietary interventions as complementary strategies for cancer prevention.

Notwithstanding these promising findings, several factors, such as genetic polymorphisms, microbiome composition, food preparation techniques, and variations in vegetable genotypes, continue to shape individual responses and pose challenges for translating preclinical results into actionable public health recommendations. Furthermore, existing clinical studies are constrained by small sample sizes, methodological heterogeneity, and limited follow-up durations. Therefore, while current evidence strongly indicates that GSLs and their derivatives hold considerable potential as natural agents for mitigating cancer risk, definitive conclusions on optimal intake levels, formulation strategies, and targeted applications require more rigorous, standardized research.

Looking to the future, research efforts should focus on the following: (i) conducting robust, placebo-controlled clinical trials to evaluate the safety and effectiveness of diets rich in glucosinolates; (ii) developing reliable biomarkers to track GSL metabolism and its physiological effects; (iii) exploring gene–diet interactions to identify population subgroups that could benefit most from consuming cruciferous foods; (iv) optimizing horticultural and postharvest practices to increase the yield and stability of beneficial compounds; and (v) developing incentives to eat CVs, such as a book of recipes and menus suitable for cancer prevention and overall wellness. In conclusion, integrating advances in plant sciences, molecular biology, and preventive medicine offers a promising approach for translational research. This strategy not only supports sustainable agriculture but also offers a food-based approach to disease prevention, underscoring the vital connection between plant science and human health.

## Data Availability

This manuscript does not report research data generation.
